# Diagnosis of Community-Acquired Pneumonia Due to Influenza or Respiratory Syncytial Virus: Evaluation of RT-PCR Sensitivity in Nasopharyngeal, Saliva, and Sputum Samples

**DOI:** 10.3390/pathogens14050400

**Published:** 2025-04-22

**Authors:** Julio Ramirez, Stephen Furmanek, Thomas Chandler, Ruth Carrico, Ashley Wilde, Alan Junkins, Anupama Raghuram

**Affiliations:** 1 Norton Infectious Diseases Institute, Norton Healthcare, Louisville, KY 40202, USA; 2 Division of Infectious Diseases, University of Louisville, Louisville, KY 40202, USA

**Keywords:** pneumonia, influenza, respiratory syncytial virus

## Abstract

Detection of viral RNA in nasopharyngeal (NP) samples by reverse transcription polymerase chain reaction (RT-PCR) is the standard diagnostic test for influenza or respiratory syncytial virus (RSV) in hospitalized patients with community-acquired pneumonia (CAP). This study compared the sensitivity of RT-PCR using NP swab, saliva, and sputum samples for the diagnosis of CAP due to influenza or RSV. A total of 60 patients were evaluated, of which 40 (67%) had influenza CAP, 19 (32%) had RSV CAP, and one patient (1%) had both RSV and influenza CAP. RT-PCR on NP swab, saliva, and sputum samples was performed using the Luminex ARIES platform. In patients with influenza CAP, the sensitivity was 34% for NP swabs, 68% for saliva, and 71% for sputum. In patients with RSV CAP, the sensitivity was 60% for NP swabs, 75% for saliva, and 85% for sputum. RT-PCR of nasopharyngeal swab samples was associated with a significant number of false negative results. A negative NP swab RT-PCR test should not be used to rule out CAP due to influenza or RSV. Saliva and sputum samples should be considered when performing a microbiological work-up in patients with suspected influenza or RSV CAP.

## 1. Introduction

Community-acquired pneumonia (CAP) remains a significant public health concern, leading to approximately 1.5 million hospitalizations annually in the United States [1]. Respiratory viruses are frequently implicated in CAP, yet a substantial proportion of cases lack an identified pathogen, despite extensive microbiologic testing [2]. A large multicenter study conducted by the Centers for Disease Control and Prevention (CDC) found that viral pathogens were the most commonly detected etiologic agents, identified in 27% of hospitalized CAP patients through viral RNA detection in nasopharyngeal (NP) samples by reverse transcription polymerase chain reaction (RT-PCR) [3]. However, no pathogen was detected in 62% of cases [3].

A meta-analysis of viral pneumonia conducted before the emergence of SARS-CoV-2 estimated that viral CAP accounted for approximately 25% of hospitalized cases, with reported incidences ranging from 9% to 56% across studies [4]. Influenza, rhinovirus, and respiratory syncytial virus (RSV) were the predominant pathogens identified [4]. Notably, this meta-analysis highlighted an increased detection rate of viral pathogens in studies utilizing lower respiratory tract samples. RT-PCR of upper respiratory specimens identified viral pathogens in 24% of patients, whereas studies incorporating lower respiratory samples detected viral etiology in more than 50% of cases [4].

One plausible explanation for this discrepancy in detection is that at the time of hospitalization, the natural history of viral CAP has progressed to the point that viral replication is primarily occurring in the lower respiratory tract. As a result, the viral load in the nasopharynx may diminish to undetectable levels, leading to false negative RT-PCR results from NP swabs. Studies of respiratory viruses in adults have demonstrated that viral RNA is often more reliably detected in sputum or tracheal aspirates than in upper airway samples, especially later in the disease course [5,6,7].

If lower respiratory tract samples are far superior to upper respiratory tract samples for the diagnosis of viral CAP, this will have significant implications in clinical practice and infection control. Missed diagnoses of viral CAP may delay pathogen-directed therapy, such as oseltamivir for influenza, and delay implementation of isolation precautions [8]. Furthermore, under-recognition of viral CAP will alter surveillance and lead to underestimation of the true burden of disease.

The extent to which RT-PCR of NP swabs may fail to correctly identify patients with viral CAP remains uncertain. Obtaining an NP swab is the current standard in clinical practice for the diagnosis of viral CAP. Since the role of alternative respiratory specimens, such as saliva or sputum, is not well defined, the best diagnostic approach for hospitalized patients with viral CAP is unclear.

Comparative studies assessing the diagnostic yield of RT-PCR across NP swabs, saliva, and sputum are limited, particularly for non-COVID-19 respiratory viruses like influenza and RSV.

The objective of this study is to evaluate the diagnostic performance of RT-PCR from NP, saliva, and sputum samples in hospitalized patients with CAP due to influenza or RSV. By comparing the sensitivity of each sample type, we aim to determine the most effective diagnostic approach for influenza or RSV CAP at the time of hospitalization.

## 2. Materials and Methods

### 2.1. Study Design and Patient Population

This study was a secondary analysis of a prospective cohort of patients hospitalized for acute respiratory illness from 27 December 2021 through 31 March 2023, across four affiliated Norton Healthcare acute care hospitals in Louisville, KY, USA [9]. Patients were eligible for enrolment in the primary study if they were (1) aged 40 years or older and (2) hospitalized with an ARI, defined as the presence of at least one of the following: (a) new onset or increase from baseline in any of following nine signs and symptoms: nasal congestion, rhinorrhea, sore throat, hoarseness, cough, sputum production, dyspnea, wheezing, or hypoxemia; (b) a diagnosis on admission suggestive of ARI; or (c) exacerbation of underlying cardiopulmonary disease involving acute respiratory symptoms. All patients enrolled consented to participate in the study. This study was approved by the Norton Healthcare Research office and the central Institutional Review Board (IRB#21-N0325).

### 2.2. Sample Collection and Processing

In the parent study, after consent, respiratory samples of NP swabs, saliva, and sputum were obtained within 72 h of arrival at the hospital. RT-PCR was performed using the Luminex ARIES platform (Luminex Corporation, Austin, TX, USA) for detection of influenza and RSV [10,11]. The Luminex ARIES platform amplifies the matrix genes for influenza A and B, as well as the fusion genes for RSV A and B [10]. The obtained samples were delivered to a central laboratory on the same day for processing. The samples were processed according to standard operating procedures [12]; additionally, sputum samples and thick saliva samples were diluted and mixed via vortex, before the mixed solution was pipetted into Luminex cartridge vials. Full details of sample processing have been published previously [9].

### 2.3. Criteria for Secondary Analysis

In the parent study, any patient with acute respiratory illness could be eligible for participation. For this secondary analysis, patients met inclusion criteria if they (1) had all three samples—NP swab, saliva, and sputum samples—obtained within 72 h of their enrollment in the study, (2) if they had a positive RT-PCR test result for either influenza or RSV for any sample type, and (3) if they had a diagnosis of CAP, defined by (A) one of the following: a new or increased cough, sputum production, or changes in WBC; (B) the presence of new pulmonary infiltrate on a chest X-ray or CT scan; and (C) no alternative diagnosis at the time of hospital discharge that explained the presence of criteria A and B.

Patients were defined as having influenza CAP or RSV CAP if any RT-PCR test result was positive for influenza or RSV, respectively.

### 2.4. Statistical Methods

Patient characteristics for the influenza CAP and RSV CAP groups were described as the mean and standard deviation for continuous variables, and the frequency and percentage for categorical variables. Venn diagrams were created to depict the overlap between sample types in detection of influenza CAP and RSV CAP.

The sensitivity of each specimen type was calculated for influenza CAP and RSV CAP. A patient was considered to have a true positive result if any sample type was positive. The sensitivity for each sample type was calculated as the number of positives from that sample type out of the true positives. Sensitivity was reported with 95% confidence intervals using the Clopper–Pearson method. Time-dependent sensitivity was also calculated by taking the time from symptom onset to sample collection. The time from symptom onset to specimen collection was described by the median and interquartile range (IQR). Analysis was performed in R version 4.4.3 [13].

## 3. Results

A total of 60 patients met the inclusion criteria for this secondary analysis: 40 patients (67%) had influenza CAP, 19 patients (32%) had RSV CAP, and one patient (1%) had both RSV and influenza CAP. The patient characteristics are summarized in Table 1. The patient with both RSV and influenza CAP was incorporated into both groups.

**Figure 1:**
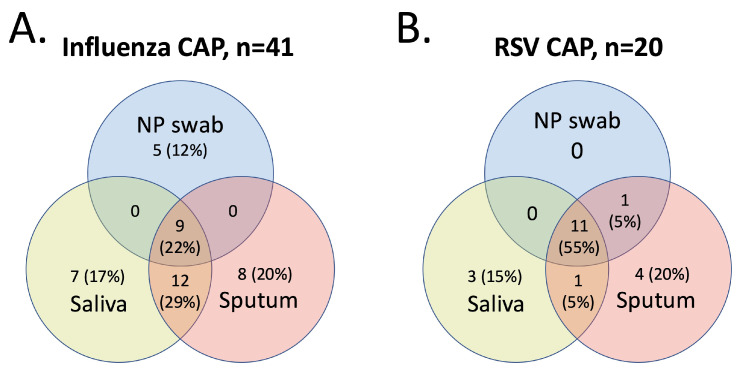
Venn diagrams of sample positivity for (**A**) detection of CAP due to influenza and (**B**) detection of CAP due to RSV.

For influenza CAP, all three samples were concordant in the detection of influenza in 9 patients (22%). For RSV CAP, all three samples were concordant in the detection of RSV in 11 patients (55%). The number of patients with influenza CAP and RSV CAP detected by each specimen type for is depicted in Figure 1.

**Figure 2:**
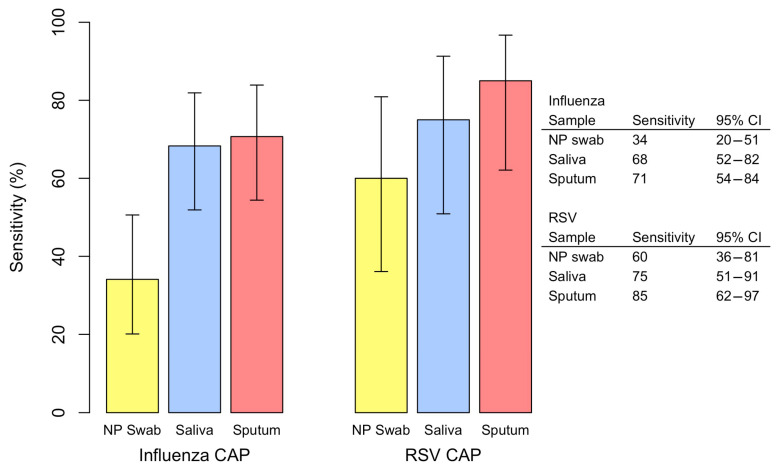
Sensitivity by sample type of detection of influenza and RSV CAP.

For both influenza CAP and RSV CAP, sputum had the highest sensitivity for detecting the microorganism. In patients with influenza CAP, the sensitivity was 34% for NP swabs, 68% for saliva, and 71% for sputum. In patients with RSV CAP, the sensitivity was 60% for NP swabs, 75% for saliva, and 85% for sputum. The sensitivity of each specimen type and the corresponding 95% confidence interval are illustrated in Figure 2.

**Figure 3:**
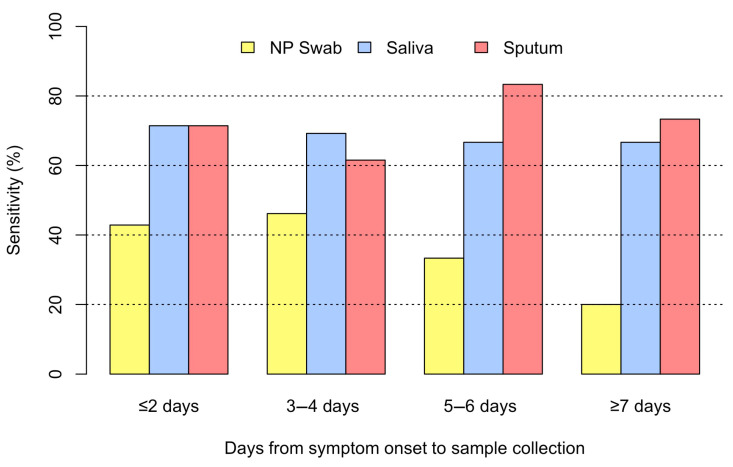
Sensitivity by timing of sample collection for influenza CAP.

The median (IQR) time from symptom onset to sample collection for CAP due to influenza was 5 (3–8) days. Figure 3 depicts the sensitivity for each specimen type in the detection of influenza among patients with CAP. The NP swab was the most sensitive when sample was collected within three to four days, saliva was the most sensitive when the sample was collected within two days, and sputum was the most sensitive when the sample was collected within five to six days after the onset of symptoms.

**Figure 4:**
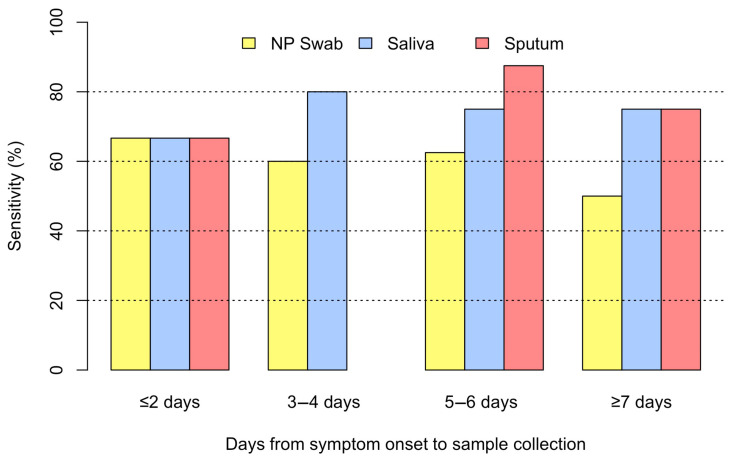
Sensitivity by timing of sample collection for RSV CAP.

The median (IQR) time from symptom onset to sample collection for CAP due to RSV was 5 (3–6) days. Figure 4 depicts the sensitivity for each specimen type in the detection of RSV among patients with CAP. The NP swab was the most sensitive when the sample was collected within two days, saliva was the most sensitive when the sample was collected within three to four days, and sputum was the most sensitive when the sample was collected within five to six days after the onset of symptoms.

As part of the standard of care in microbiological work-ups, the following organisms were identified. In patients with RSV CAP, one patient (5%) had coinfection with SARS-CoV-2. In patients with influenza CAP, four patients (10%) had coinfection with *Streptococcus pneumoniae* and two patients (5%) had coinfection with SARS-CoV-2.

## 4. Discussion

This study indicates that RT-PCR of nasopharyngeal swabs failed to detect a significant number of hospitalized patients with influenza or RSV CAP in whom saliva or sputum samples yielded positive results. Nasopharyngeal swab samples detected only 34% of patients with influenza CAP and 60% of patients with RSV CAP. Sputum samples offered the highest sensitivity for detecting either virus, with 85% sensitivity for detecting RSV CAP and 71% sensitivity for detecting influenza CAP.

The use of saliva and sputum as a respiratory specimen for the detection of influenza and RSV in adult hospitalized patients has been previously reported in the literature. In a study of 214 patients from Hong Kong, the investigators concluded that saliva specimens had high sensitivity and specificity in the detection of respiratory viruses [14]. In a study of 154 patients from Korea, the investigators concluded that the detection rates of respiratory viruses from sputum were significantly higher than those from NP swabs, with 27% of influenza infections and 41% of RSV infections detected only in sputum samples [15].

Fluid from bronchoalveolar lavage (BAL) is considered the gold-standard sample for use in the etiologic diagnosis of CAP. In a study of 276 patients from France hospitalized with CAP, the investigators evaluated the concordance of PCR detection performed on NP swabs and BAL fluid [16]. Out of 13 patients with BAL fluid positive for RSV, the NP swab was positive in 8 patients (61.5%). Out of 24 patients with BAL fluid positive for influenza, the NP swab was positive in 19 patients (79%). Among all 95 patients with a positive result based on BAL fluid, 68 were also positive based on NP swabs. The sensitivity of NP swabs was 71.6% (95% CI 61.4–80.4%).

Similar findings have been reported from the UK for hospitalized patients with SARS-CoV-2 CAP requiring ICU care [17]. In this study, results from NP swabs were compared to those from lower respiratory samples, such as sputum, tracheal aspirate, or BAL fluid, in 52 patients. NP swab samples detected SARS-CoV-2 in only 67% of patients.

In our study, we evaluated sample positivity in relation to the duration between the onset of symptoms and sample collection. We identified a trend of decreasing positivity in NP swab detection of influenza and RSV with an increasing number of days from symptom onset to sample collection. These findings support the concept that in some hospitalized patients with CAP, the initial viral replication in the nasopharynx may decrease during hospitalization to an undetectable level, and lower airway viral multiplication may be predominant. Our findings are consistent with the pathophysiology of pneumonia, in that viral replication occurs primarily in the alveolar space. As a result, lower respiratory samples have a higher viral load, which may translate into higher detection on RT-PCR.

The strengths of this study include the exclusion of patients for whom not all three sample types were obtained. Additionally, these specimens were obtained from patients who represented a typical patient hospitalized with CAP, with a comparable age and comorbidity burden. Important limitations of our study include the small sample size and the evaluation of only two respiratory viruses. These limitations may restrict the generalizability of our findings.

The current standard of practice for using NP swabs for the diagnosis of respiratory viruses emerged from the pediatric literature. In children, NP swabs provide a standardized and reproducible sample from a well-defined anatomical site. Sputum is difficult to obtain, since children often cannot expectorate sputum. Saliva production is inconsistent, and collection of a saliva sample requires patient cooperation, which can be challenging in young children. In contrast to children, saliva and expectorated sputum samples are more easily obtained from adults with pneumonia. An NP swab is often described as an uncomfortable test in adults. Other advantages of saliva or sputum samples in adults are that sample collection does not require a trained healthcare professional. Based on the evidence in the literature, the results of our study, and the simplicity of sample collection, obtaining saliva and sputum samples for influenza or RSV detection should be strongly considered in hospitalized patients with CAP.

## 5. Conclusions

In hospitalized patients with influenza or RSV CAP, RT-PCR of NP swab samples is associated with false negative results. The possibility of CAP due to influenza or RSV should not be ruled out based solely on a negative NP swab RT-PCR test. Saliva and sputum samples should be considered when performing a microbiological work-up in patients with suspected influenza or RSV CAP.

## Figures and Tables

**Table 1:** Participant demographics, comorbidity history, and severity of disease for influenza and RSV CAP.

Variable	Influenza CAP *n* = 41	RSV CAP *n* = 20
Age in years, mean ± SD	63.9 ± 11.3	63.6 ± 12.0
Male sex, *n* (%)	24 (59%)	10 (50%)
White or Caucasian race, *n* (%)	30 (73%)	14 (70%)
Black or African American race, *n* (%)	8 (20%)	5 (25%)
Other race, *n* (%)	3 (8%)	1 (5%)
History of neoplastic disease, *n* (%)	5 (12%)	1 (5%)
History of COPD, *n* (%)	18 (44%)	8 (40%)
History of CHF, *n* (%)	11 (28%)	8 (40%)
History of stroke, *n* (%)	2 (5%)	0 (0%)
History of renal disease, *n* (%)	4 (10%)	5 (25%)
History of liver disease, *n* (%)	4 (10%)	0 (0%)
History of diabetes, *n* (%)	13 (32%)	10 (50%)
PSI risk class IV/V	9 (22%)	5 (25%)

Abbreviations: CAP: community-acquired pneumonia; RSV: respiratory syncytial virus; SD: standard deviation; COPD: chronic obstructive pulmonary disease; CHF: congestive heart failure; PSI: pneumonia severity index.

## Data Availability

Data are available upon request.

## Abbreviations

The following abbreviations are used in this manuscript:

CAP	Community-acquired pneumonia
RSV	Respiratory syncytial virus
RT-PCR	Reverse transcription polymerase chain reaction
NP	Nasopharyngeal
BAL	Bronchoalveolar lavage
